# Heading towards the Safer Highways: an assessment of the Avahan prevention programme among long distance truck drivers in India

**DOI:** 10.1186/1471-2458-11-S6-S15

**Published:** 2011-12-29

**Authors:** Arvind Pandey, Ram Manohar Mishra, Damodar Sahu, Sudhir Kumar Benara, Uttpal Sengupta, Ramesh S Paranjape, Abhishek Gautam, Satya Ranjan Lenka, Rajatshurva Adhikary

**Affiliations:** 1 National Institute of Medical Statistics, New Delhi, India; 2 Population Council, New Delhi, India; 3 National AIDS Research Institute, Pune, India; 4 Family Health International, New Delhi, India

## Abstract

**Background:**

Using data from two rounds of a cross-sectional, national-level survey of long-distance truck drivers, this paper examines the extent and trend of sexual risk behavior, prevalence of STI/HIV, and the linkage between exposure to HIV prevention programs and safe sex behavior.

**Methods:**

Following the time location cluster sampling approach, major transshipment locations covering the bulk of India’s transport volume along four routes, North-East (NE), North-South (NS), North-West (NW) and South-East (SE) were surveyed. First round of the survey was conducted in 2007 (sample size 2066) whereas the second round was undertaken in 2009-2010 (sample size 2085). Long distance truck drivers were interviewed about their sexual behaviors, condom use practices, exposure to different HIV prevention interventions, and tested for HIV, reactive syphilis serology, *Neiserria gonorrhoeae* and *Chlamydia trachomatis.* The key variable of this evaluation study - exposure to HIV prevention interventions was divided into three categories - no exposure, less intensive exposure and intensive exposure. Data were analyzed using multiple logistic regression methods to understand the relationship between risk behavior and exposure to intervention and between program exposure and condom use.

**Results:**

The proportion of truckers exposed to HIV prevention interventions has increased over time with much significant increase in the intensive exposure across all the four routes (NE: from 14.9% to 28%, P < 0.01; NS: from 20.9% to 38.1%; NW: 11.5% to 39.5%, P < 0.01; SE: 4.7% to 9.7%, P <0.05). Overall, the consistent condom use in sex with non-regular female partners too has increased over the time (paid female partners: from 67.1% to 73.2%, P <0.05; non-paid female partners: from 17.9% to 37.1%, P <0.05). At the aggregate level, the proportion tested HIV positive has declined from 3.2% to 2.5% in (p<0.10) and proportion tested positive for Syphilis too has reduced from 3.2% to 1.7% (p<0.05). Truckers who had sex with paid female partners (men at risk) were significantly more likely to get exposed to intensive program (aOR: 2.6, 95%CI 1.9-3.4) as compared to those who did not have sex with paid partners. Truckers who had sex with paid partners and exposed to intervention program were more likely to use condoms consistently (aOR: 2.1, 95% CI 1.2-3.7). The consistent condom use among respondents who travel through states with targeted interventions towards female sex workers was higher than those who travel through states with less intensive program among FSWs.

**Conclusions:**

These evaluation study results highlight the ability of intensive program to reach truckers who have sex outside marriage with HIV prevention interventions and promote safe sex behaviors among them. Truckers who practice safe sex behaviors with an exposure to intensive program are less likely to suffer from STIs and HIV, which has implications for HIV prevention program with truckers’ population in India and elsewhere. The simultaneous targeted interventions among female sex workers appeared to have contributed to safe sexual practices among truckers.

## Introduction

India trucking population is estimated at five to six million truck drivers and helpers, with about two to two and half million being long-distance truckers [1]. The Indian long-distance trucking industry consists of three different segments: free agents, port operators, and express cargo operators. Truckers tend to specialize in any one of these segments, primarily because it is difficult to build business networks in more than one segment. The free agent segment which accounts for approximately 70 percent of the long-distance truckers, is fragmented with a vast majority working for small transport operators [2]. In the late 1990s, almost 77% of India’s truck fleet was owned by operators with no more than five trucks, while only about 6% of trucks were owned by operators with more than 20 trucks [3]. This ownership profile created middlemen (transporters and brokers) on whom small trucking operators depend to generate business. This structure of the Indian trucking industry has diluted the visibility of the industry to transport planners and policy-makers [4,5].

Truck drivers and their helpers, particularly those who travel on highways for longer distances, have been associated with the spread of sexually transmitted infections including HIV in many parts of the world including India [6-18]. Long distance truckers are considered to be particularly vulnerable to STIs and HIV infection because they spend many days away from their families in contrast to short-distance, state-level truckers [7,18]. Earlier reports demonstrated that in spite of high rates of STI prevalence of HIV remained lower in long distance truck drivers [19,20]. However, because of high-risk behavior coupled with their mobility these long distance truckers are said to have potential of spreading HIV to different geographical areas [1,7,9]. For these reasons, truckers have been key target populations in the Indian national response since 1996 under National AIDS Control Program II and III. The three major components of the target interventions among truckers are- (1) Behavioral Change Communication (2) Condom promotion activity through social marketing and free distribution of condoms, and (3) Treatment of sexually transmitted infections (STIs) [21,22]. Given that HIV programs in India are implemented at the state level through State AIDS Control Societies, state-level truckers were routinely covered under Targeted Interventions funded by the National AIDS Control Organization. However, long distance truckers who worked on the national highways were not targeted specifically in these interventions [23,24].

In 2003, Avahan, the India AIDS Initiative was started in India with the aim to slow down the HIV epidemic through focused, integrated, large-scale prevention programs providing saturated coverage to high risk populations including female sex workers (FSW), men who have sex with men, transgenders, injecting drug users in the six high prevalence states in India. Potential clients of FSWs were also targeted through interventions at sex worker solicitation areas (“hot-spots”) along with long distance truck drivers (LDTD) [24,25]. The Avahan intervention with LDTD began in 2004. To enhance accessibility of clinical services to truckers *Khushi* (meaning ‘happiness’ in Hindi/Urdu) clinics were established at 36 truck halt points. This intervention was redesigned in 2006 by halving the numbers of implementation sites from 36 to 17 focusing on the major truck halt points in nine Indian States. It was revamped to take advantage of the structure of the Indian trucking industry with middlemen where truckers spend time between shipments. It recruited peer educators, increased the emphasis on professional media expertise in mid-media and mass media events, improved signage and satellite clinical services at the halt points. More details about the interventions can be found elsewhere [2,25].

A component of the evaluation design of the Avahan intervention includes two rounds of large scale cross-sectional surveys of long distance truck drivers with both a behavioral and biological component [26]. The two rounds of the surveys are known as Integrated Behavioral and Biological Assessment (IBBA) on national highways. This paper presents an analysis of both rounds of IBBA on national highways to assess whether highways have become ‘safer’ in terms of risk of HIV transmission among truckers. By safer highways we mean increase in exposure to HIV prevention interventions and consistent condom use with non regular female sexual partners along with reduction in sexually transmitted infections (STI) including HIV among truckers. As the IBBA on national highways was conducted only among long distance truck drivers, the term trucker in this paper stands for long distance truck drivers (and not the helpers).

## Data and methods

The first round of IBBA on national highways was conducted in 2007 at seven transshipment locations (TSL) covering the bulk of India’s transport volume along four routes, North-East (NE), North-South (NS), North-West (NW) and South-East (SE). TSL were the places where the transporters and brokers operate by linking truckers with individuals wanting their goods to be transported and the route categories were the road corridors traveled by LDTD. Following TSL were considered as survey sites- Sanjay Gandhi Transport Nagar, New Delhi; Ghaziabad Transport Nagar, Ghaziabad; Kalamboli, Mumbai; Narol Chowkdi, Ahmedabad; Gandhidham, Kandla; Neelamangala, Bangalore and Territy bazar, Kolkata. The first survey round covered a total of 2,066 long distance truck drivers (NE- 498; NS- 540; NW- 515; SE- 513) with an overall participation rate of 97% (NE- 97%; NS- 96%; NW- 98% and SE- 98%). Results from the first round of survey are available [17].

**Figure 1:**
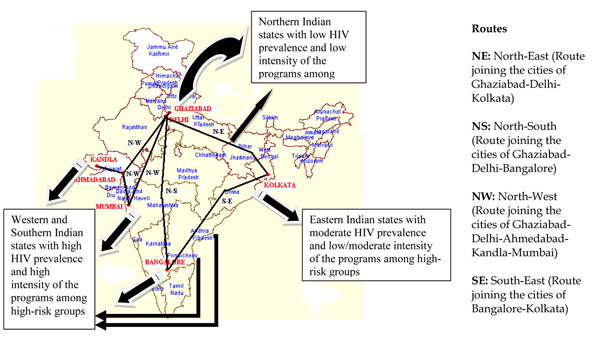
Route Corridors Covered by Avahan Intervention Sampled in IBBA on National Highways

The second round of the survey was conducted in 2009-10 at same TSL except for that in Kandla along the four aforesaid routes. The main reason behind excluding TSL at Kandla was less availability of long distance truck drivers. The TSL and route categories covered in both rounds are shown in Figure 1. Identical survey design and methodology as described below were adopted in both survey rounds.

### Methods

A sample size of 500 long-distance truck drivers was used for each route. A two-stage time-location cluster sampling approach was used to select the respondents. Behavioral data were collected by face to face interview using a pre-tested, pre-coded questionnaire translated into local languages by native speakers. It collected information on demographics, work, mobility, sexual behavior, history and symptoms of STI, knowledge of HIV and its prevention and exposure to HIV prevention interventions supported by Avahan and other agencies.

Blood and urine samples were collected from all participating truckers. Anticubital venipunctured blood sample (5ml) collected in a vacutainer was allowed to clot for separation of serum and was stored at 2° to 8°C. From each participant 30 ml urine sample was collected and from this 2 ml quantity only was stored in a urine specimen transport tube as per the protocol of M/s Gen –Probe Aptima Combo 2 Assay (Gen-Probe Incorporated, USA). Sera were tested for both HIV-1 and HIV-2 by Microlisa HIV kit (J. Mitra & Co. Pvt Ltd, India and GENEDIA HIV ½ ELISA 3.0 Kit, Gencross Life Science Corporation, Korea). Syphilis reactive serology was performed by Rapid Plasma Reagin Test Kit (Span Diagnostics Ltd, India) and was confirmed by *Teponema pallidum* hemagglutination assay (TPHA) using Syphagen TPHA Kit). All cases with RPR reactive serology of any titer with TPHA positivity were considered positive. For the diagnosis of *Neisseria gonorrhoeae* and *Chlamydia trachomatis*, urine samples were tested using Transcription- Mediated Amplification Assay and Dual Kinetic Assay (Gen-Probe Incorporated, USA). About 10 percent of serum samples were screened for herpes simplex virus type 2 using HerpeSelect 2 ELISA IgG Kit (FOCUS Technologies, USA).

The study was approved by all relevant institutional review boards (Health Ministry Screening Committee, Government of India, Scientific Advisory Committee of National AIDS Research Institute, Protection of Human Subjects Committee of Family Health International and Scientific Advisory Committee and Ethical Committee of National Institute of Medical Statistics). Participation followed written informed consent and all data were recorded in a linked anonymous manner using numerically coded cards. Clinics run by the Transport Corporation of India Foundation at highway locations were used to enable participants to obtain syphilis test results and treatment upon presentation of the numerically coded cards. More information about the survey methodology can be found elsewhere [17,26,27].

## Measures

### Programme exposure

Based on information from truckers on their awareness of HIV prevention interventions and utilization of services from Avahan or non-Avahan interventions three categories of program exposure were created: no program exposure if they never heard of any HIV prevention intervention along their route; less intensive exposure if they heard of HIV prevention intervention but either did not utilize its services in past 12 months, or received services only from non-Avahan interventions; intensive exposure if they received any of the following services either from Avahan or from both Avahan and non-Avahan at least once in past 12 months- contacts by peer educators/ out-reach workers, receipt of condoms from peer educator or outreach worker, visit to Khushi clinics, counseling services on HIV/AIDS, , participation in any community meeting or events (such as street plays, health games, truckers’ festival) organized by Khushi clinic. By non-Avahan interventions we mean all interventions which are being implemented by agencies other than Avahan. Our definition of classifying interventions under Avahan as being more intensive than interventions under other program is consistent with that provided elsewhere [25,28].

### Sexual behaviors

Two types of non-regular female sexual partners were defined- paid female partners (PFP) and non-paid female partners (NPFP). The paid female partner was defined as women whom the respondent had paid cash in exchange for having sex in past 12 months. A non-paid female partner was defined as women with whom the responded had sex but was not marred to and did not pay cash in exchange for sex in past 12 months. These variables were derived based on the single questions asked in the questionnaire. Age at first paid sex was defined as age of the respondent at first paid sex and was also derived based on a single question asked in the questionnaire.

### Consistent condom use

Consistent condom use with both paid and non-paid female sex partners was the primary outcome variable measuring HIV-related risk behaviour. The consistent condom use with a given sexual partner was defined as use of condom in every sexual encounter with that particular partner in past 12 months.

### Socio-demographic and work related characteristics

The socio-demographic characteristics include their age (in completed years), literacy (the ability to both read and write) and marital status (currently married verses not). The work related characteristics are route category on which the respondent usually travels, years of working as a driver, number of round trips between main cities of operation in past six months and ownership of trucks by respondent (yes verses no). These variables were derived based on single questions asked in the questionnaire.

These abovementioned socio-demographic and work related characteristics along with variable measuring age at first paid sex were controlled in all multivariate analyses done in the paper.

### Prevalence of STI/HIV

Prevalence of following four STIs is given- Syphilis, *N. gonorrhoeae* and *C. trachomatis* and HIV. By Syphilis, we mean active Syphilis infection. Any STI was defined as presence of at least one of the four above said STIs. As HSV-2 was tested only among 10% of the subsample, it has not been considered for the analysis. A respondent was termed as HIV positive if he was found positive either for HIV-1 or HIV-2 or both.

### Statistical techniques

For both rounds of the survey two sets of sampling weights were computed; one route specific for getting route-wise estimates and the other was overall weights for combined dataset. By combined dataset we mean data obtained by combining route-specific data. Statistical software Stata (version 11) was used for statistical analysis.

We have done route-wise analysis using data from both rounds of the survey to provide estimates related to socio-demographic and work related characteristics, program exposure, sexual behavior and prevalence of STI/HIV at two points of time. Differences in the mean values between the survey rounds were tested using the t-test while differences in the percentages were tested using z-test statistic. To examine the association of program exposure with risk behavior we have used data from second round only. It has been done because the first round was considered as proxy for the baseline [17]. Moreover, the interventions (particularly those belonging to Avahan) were in place for substantial duration in 2009-10 when second round of the survey was undertaken.

Cross-tabulations were made to examine the association between having sex with non-regular female partners and program exposure. Differences between the categories were tested using Chi-square test. A multivariate multinomial logistic regression analysis with program exposure as dependent variable was done to examine this association after adjusting for the control variables. Similarly, to examine the associations between program exposure and consistent condom use with non-regular partners, cross-tabulations were made followed by two multivariate logistic regressions with following two outcome variables- (1) consistent condom use with paid female partners in past 12 months (ii) consistent condom use with non-paid female partners in past 12 months. While the bi-variate analyses were carried out separately for each route the multivariate analysis were restricted to combined data only.

## Results

In second round of the IBBA on national highways, a total of 2085 truckers for whom both behavioral and biological data were available were used for analysis (NE- 524; NS- 538; NW- 526; SE- 497). The overall participation rate in second round of the survey was 77% (NE- 76%; NS- 74%; NW- 85% and SE- 71%).

### Background characteristics

Table 1 depicts the background characteristics and reported program exposure at two points of time among long distance truck drivers who travel on the four routes. The mean ages of respondents at the aggregate level as well as in NE and NW route were significantly increased between the two survey rounds. Literacy was increased across all the four routes and the differences were significant in NE, NS and SE route. Percentage of drivers who own the truck was significantly reduced on NE (from 8.9% to 4.8%, P <0.05) and NS (from 17.1% to 2.4%, P <0.05) routes between the two rounds. No significant differences were observed in the marital status of the respondents and average number of years of working as drivers. The average number of round trips between main cities of operation in past six months remained almost constant over time except for NW route where it declined significantly from 13.4 to 11.2 (P<0.05).

**Table 1:** Background Characteristics and Reported Exposure to Interventions among Long Distance Truck Drivers Round-1 and Round-2 of the IBBA on National Highways

	All Routes		North-East (NE)		North-South (NS)		North-West (NW)		South-East (SE)	
	
Background characteristics	Round-1 (N= 2066)	Round-2 (N= 2085)	Round-1 (N= 498)	Round-2 (N= 524)	Round-1 (N= 540)	Round-2 (N= 538)	Round-1 (N= 515)	Round-2 (N= 526)	Round-1 (N= 513)	Round-2 (N= 497)
Average Age	31.1	33.5***	31.8	33.4*	31.4	31.8	30.1	32.4**	33.9	35.0
Percent literates	87.6	94.1**	83.3	89.7*	90.8	97.5**	88.5	89.6	87.3	94.6**
Percent Currently married	73.5	74.3	75.2	74.2	74.9	71.2	72.0	73.6	82.3	84.0
Average number of years of working as a driver	8.9	9.4	9.7	9.7	9.3	9.9	8.0	8.5	10.7	11.1
Average number of round trips in past six months^a^	11.9	11.2	11.4	11.1	10.1	10.1	13.4	11.2**	12.4	12.7
Percent who own truck	14.2	9.0***	8.9	4.8**	13.9	13.5	17.1	2.4**	13.1	11.2
**Exposure to interventions (%)**										
No	49.8	45.0**	44.4	48.9	36.7	42.8	61.0	36.4***	34.7	62.7***
Less intensive	36.1	25.4***	40.7	23.1***	42.4	19.2	27.5	24.1	60.2	27.6***
Intensive	14.1	29.6***	14.9	28.0***	20.9	38.1**	11.5	39.5***	4.7	9.7**

* P< 0.10; ** P< 0.05; *** P <0.01

Differences between the mean values were tested using t-test whereas the differences between percentages were tested using Z-test statistic.

Averages refer to the mean values.

Literacy refers to the ability of both read and write.

^a^ Between main cities of operation.

### Trends in exposure to intensive HIV prevention program

Overall, the percentage of respondents with no program exposure declined between two survey rounds. This percentage remained almost same for NE and NS route whereas it decreased significantly on NW route (from 61.0% to 36.4%; P < 0.01) and increased significantly on SE route (from 34.7% to 62.7%; P<0.01). The intensive program coverage was significantly increased across all the four routes between the two rounds of survey (NE: from 14.9% to 28.0%, P<0.01; NS: from 20.9% to 38.1%, P<0.05; NW: from 11.5% to 39.5%, P<0.01; SE: from 4.7% to 9.7%, P<0.05). On the other hand, less intensive exposure was declined significantly on NE and SE routes and remained at constant level in the remaining two routes.

### Trend in risk behavior and STI/HIV prevalence

Table 2 provides the trend in risk behavior and STI/HIV prevalence among respondents. In both survey rounds about one-quarter to one-third of the long distance truck drivers on the NE, NS and NW routes reported having had sex with a paid female partner. In contrast, the percentage of reported sex with paid female partners dropped significantly on SE route, from 44% to 14% (P <0.05). Overall, there was a significant increase in the mean age at first paid sex between two rounds. These were significantly increased over time on NW and NS routes respectively. A significant increase in reported consistent condom use with paid female partners was noticed in NW (from 60.5 to 75.8%; P <0.05) and SE route (from 63.8 to 87.5%; P <0.05). The proportion of long distance truck drivers who reported visiting non-paid female partners in the past one year was significantly increased in NE and NW routes whereas it remained at almost same level in other routes. A significantly higher consistent condom use with non-paid female partners was reported in three out of four routes- NS (from 20.4% to 49.1%, P<0.05), NW (from 15.3% to 32.6%, P <0.10) and SE (from 14.0% to 31.6%, P <0.10).

**Table 2:** Trend in Sexual Behavior and STI/ HIV Prevalence among Long Distance Truck Drivers between Round-1 and Round-2 of the IBBA on National Highways

	All Routes		North-East (NE)		North-South (NS)		North-West (NW)		South-East (SE)	
	
Sexual Behavior	Round-1	Round-2	Round-1	Round-2	Round-1	Round-2	Round-1	Round-2	Round-1	Round-2
Had sex with PFP in past one year (N)	31.0 (2066)	24.3** (2085)	24.8 (498)	25.6 (524)	30.3 (540)	28.9 (538)	29.1 (515)	28.6 (526)	43.7 (513)	13.9*** (497)
Mean age at first paid sex (N)	21.0 (640)	22.3** (507)	21.4 (124)	22.4 (134)	20.6 (164)	22.1*** (155)	21.5 (150)	22.9* (149)	20.9 (224)	21.7 (69)
Consistent condom use with PFP in past one year (N)	67.1 (640)	74.6** (507)	68.5 (124)	64.5 (134)	71.4 (164)	75.6 (155)	60.5 (150)	75.8** (149)	63.8 (224)	87.5** (69)
Had sex with NPFP in past one year (N)	20.4 (2066)	19.6 (2085)	17.8 (498)	23.2* (524)	21.6 (540)	17.1 (538)	21.7 (515)	34.1** (526)	20.9 (513)	15.3 (497)
Consistent condom use with NPFP in past one year (N)	17.9 (421)	37.1** (423)	18.9 (89)	33.7 (122)	20.4 (117)	49.1** (92)	15.3 (112)	32.6* (179)	14.0 (107)	31.6* (76)
**STI/HIV** (N)	(2066)	(2085)	(498)	(524)	(540)	(538)	(515)	(526)	(513)	(497)
Syphilis	3.2	1.7**	3.7	3.7	3.2	1.3*	3.0	1.3*	1.2	0.2*
N. Gonorrhoeae^	0.3	0.1	0.3	0.3	0.7	0.0	0.0	0.0	0.0	0.0
C. Trachomatis^	0.3	0.7	0.9	0.7	0.0	0.6	0.0	0.7	0.6	0.9
HIV	3.5	2.5*	3.1	2.5	2.4	2.2	3.8	1.9*	6.8	3.3*
Any STI	6.9	4.7**	7.6	6.9	6.1	3.7*	6.5	3.8*	8.2	4.3**

* P< 0.10; ** P< 0.05; *** P <0.01

Differences between the mean values were tested using t-test whereas the differences between percentages were tested using Z-test statistic.

PFP: Paid female partner; NPFP: Non-paid female partner

^Statistical tests were not applied for because of low prevalence.

Any STI refers to presence of at least one of the four STIs- Syphilis, N. Gonorrhoeae, C. Trachomatis and HIV.

Overall, the Syphilis prevalence was declined among long distance truck drivers from 3.2% to 1.7% (P<0.05). This decline was visible in three out of four routes- NS (from 3.2 to 1.3%; P <0.10), NW (from 3.0 to 1.3%; P <0.10) and SE (from 1.2 to 0.2%; P <0.05). *N. gonorrhoeae* and *C. trachomatis* remained at low constant level over time. At the aggregate level, a decline in the HIV prevalence from 3.5 to 2.5% (P< 0.10) was noticed among long distance truck drivers between two survey rounds. The same was true for NW (from 3.8 to 1.9%; P <0.10) and SE (from 6.8 to 3.3%; P <0.10) route where HIV decline was noticeable. A significant decline in the prevalence of any STI (from 6.9 to 4.7%; P < 0.05) was observed at aggregate level. The same trend was observed in three of the four routes- NS (from 6.1 to 3.7%; P <0.10); NW (from 6.5 to 3.8%; P <0.10) and SE (from 8.2 to 4.3%, P <0.05).

### Association between sex with non-regular female partners and program exposure

Table 3 describes the bi-variate association between having sex with paid and non-paid female partners and program exposure. The program exposure was positively associated with having sex with paid female partners in past 12 months across all the four routes. These associations were statistically significant in NE and NW routes as well as at aggregate level. Similarly, program exposure was positively linked with having sex with non-paid female partners in past 12 months. However, the association could reach at statistical significance only in NW route.

**Table 3:** Associations between sex with paid and non-paid female partner and exposure to the program, IBBA Round-2 on National Highways

	Exposure to HIV prevention program		
	**% not exposed**	**% exposed to less intensive program**	**% exposed to intensive program**

**All Routes**			
Had sex with PFP in last 12 months*** No (N= 1578) Yes (N = 507)	50.6 26.5	24.2 29.5	25.2 44.0
**Had sex with NPFP in last 12 months** No (N= 1618) Yes (N = 467)	44.7 46.0	24.7 28.5	30.6 25.5
**North-East**			
Had sex with PFP in last 12 months *** No (N= 387) Yes (N = 137)	56.1 28.2	22.1 26.0	21.8 45.8
**Had sex with NPFP in last 12 months** No (N= 387) Yes (N = 137)	50.0 45.4	22.6 24.7	27.4 29.9
**North-South**			
**Had sex with PFP in last 12 months** No (N= 390) Yes (N = 148)	43.5 41.0	18.7 20.3	37.8 38.7
**Had sex with NPFP in last 12 months** No (N= 443) Yes (N = 95)	42.1 45.7	17.6 26.7	40.2 27.3
**North-West**			
Had sex with PFP in last 12 months** No (N= 369) Yes (N = 157)	39.8 27.9	26.7 16.9	33.2 55.2
Had sex with NPFP in last 12 months** No (N= 356) Yes (N = 170)	42.7 24.2	17.9 36.1	39.4 39.7
**South-East**			
**Had sex with PFP in last 12 months** No (N= 432) Yes (N = 65)	63.9 55.6	27.8 26.9	8.4 17.5
**Had sex with NPFP in last 12 months** No (N= 432) Yes (N = 65)	67.7 61.8	30.0 27.2	2.2 10.9

** P< 0.05; *** P <0.01

Differences between the categories were tested by using Chi-square test.

PFP: Paid female partner; NPFP: Non-paid female partner

Results from multinomial logistic regression confirmed these bi-variate associations of having sex with paid female partners (Table 4) and non-paid female partners (Table 5). Table 4 depicts that those who had sex with paid female partners in past 12 months were significantly more likely to have either less intensive (aRRR = 2.5, 95%CI 1.9 – 3.5) or intensive program exposure (aRRR = 3.8, 95%CI 2.8– 4.9) as compared to those who did not have paid sex in same duration. Similarly Table 5 shows that those who had sex with non-paid female partners in past 12 months were significantly more likely to have less intensive program (aRRR = 1.6, 95%CI 1.2-2.5) than their counterparts. However, in this case exposure to intensive program was not found to have any significant association.

**Table 4:** Multivariate Analysis: Exposure to intervention and sex with paid female partners, IBBA Round-2 on National Highways

Sex with paid female partners	Exposure to HIV prevention program	
	
	Less intensive exposure vs. No exposure	Intensive exposure vs. No exposure
	
	Adjusted RRR [95% CI]	Adjusted RRR [95% CI]
**Had sex with PFP in last 12 months** No (Reference category) Yes	1.0 2.5 [1.9 – 3.5]	1.0 3.8 [2.8 – 4.9]
**Route Categories** North-East (Reference category) North-South North-West South-East	1.00 1.2 [0.9 – 1.6] 1.2 [0.8 – 1.8] 0.9 [0.6 – 1.1]	1.00 2.1 [1.6 – 2.7] 1.9 [1.3 – 2.7] 0.2 [0.2 – 0.5]
Current age (years) ^a^	1.0 [0.9 – 1.1]	0.9 [0.9 – 1.0]
**Literacy** Illiterate (Reference category) Literate	1.0 1.4 [0.9 -2.1]	1.0 2.6 [1.5 - 4.6]
**Marital status** Not currently married (Reference category) Currently married	1.0 1.5 [1.1 -1.9]	1.0 2.5 [1.6 -2.9]
Duration of working as truck driver (years) ^a^	1.0 [0.9 – 1.1]	1.1 [0.9 – 1.2]
Number of round trips ^a, b^	0.9 [0.8 – 1.2]	1.0 [0.9 – 1.1]
**Ownership of truck**		
Respondent (Reference category) Other	1.0 0.8 [0.6 -1.3]	1.0 0.5 [0.3 -1.0]

RRR: Relative Risk Ratio; PFP: Paid female partner; NPFP: Non-paid female partner; 95% CI: 95% Confidence Interval

The analysis was done using combined data from all four routes. Adjusted RRR were estimated using multivariate multinomial logistic regression models.

^a^ Entered as continuous variable in the multivariate multinomial logistic regression models.

^b^ Between main cities of operation in past six months

**Table 5:** Multivariate Analysis: Exposure to intervention and sex with non-paid female partners, IBBA Round-2 on National Highways

Sex with non-regular female partners	Exposure to HIV prevention program	
	
	Less intensive exposure vs. No exposure	Intensive exposure vs. No exposure
	
	Adjusted RRR [95% CI]	Adjusted RRR [95% CI]
**Had sex with NPFP in last 12 months** No (Reference category) Yes	1.00 1.6 [1.2 – 2.5]	1.00 1.1 [0.8 – 1.4]
**Route Categories** North-East (Reference category) North-South North-West South-East	1.00 1.2 [0.9 – 1.6] 1.1 [0.8 – 1.7] 0.8 [0.6 – 1.0]	1.00 1.9 [1.5 – 2.5] 1.8 [1.3 – 2.8] 0.2 [0.1 – 0.4]
Current age (years) ^a^	1.0 [0.9 – 1.1]	0.9 [0.9 – 1.1]
**Literacy** Illiterate (Reference category) Literate	1.0 1.3 [0.9 -2.1]	1.0 2.6 [1.5 - 4.6]
**Marital status** Not currently married (Reference category) Currently married	1.0 1.2 [0.9 -1.8]	1.0 1.7 [1.2 -2.4]
Duration of working as truck driver (years) ^a^	0.9 [0.8 – 1.0]	1.0 [0.9 – 1.0]
Number of round trips ^a, b^	1.0 [0.9 – 1.2]	1.1 [0.8 – 1.2]
**Ownership of truck**		
Respondent (Reference category) Other	1.0 0.8 [0.6 -1.3]	1.0 0.7 [0.4 -1.1]

RRR: Relative Risk Ratio; PFP: Paid female partner; NPFP: Non-paid female partner; 95% CI: 95% Confidence Interval

The analysis was done using combined data from all four routes. Adjusted RRR were estimated using multivariate multinomial logistic regression models.

^a^ Entered as continuous variable in the multivariate multinomial logistic regression models.

^b^ Between main cities of operation in past six months

### Association between program exposure and safe sex behavior

Table 6 shows the associations between program exposure and consistent condom use with paid and non-paid female partners. Overall, those exposed to intensive program were more likely to use condom consistently with paid female partners than those who were not exposed to any of the HIV prevention program (83.0% versus 63.9%; OR = 2.7, 95%CI 1.7 – 4.6). The same relationship was found in three out of four routes- NE, NS and SE. At the same time, intensive program exposure did not have any association with the consistent condom use with non-paid female partners. On contrary, those exposed to less intensive program were more likely to use condom consistently use with non-paid female partners than those who were not exposed to any of the HIV prevention program (52.6% versus 33.9%; OR= 2.2, 95%CI 1.3 – 3.4). The same relationship was found in three out of four routes- NE, NW and SE. However, exposure to less intensive exposure could not establish any significant association with consistent condom use with paid female partners.

**Table 6:** Association between exposure to intervention and consistent condom use with paid and non-paid female partners, IBBA Round-2 on National Highways

Exposure to HIV prevention program	Consistent condom use with PFP			Consistent condom use with NPFP		
	
	N	%	Crude OR [95% CI]	N	%	Crude OR [95% CI]
**All Routes**						
No	174	63.9	1.0	217	33.9	1.0
Less intensive	107	71.6	1.4 [0.8 – 2.3]	104	52.6	2.2 [1.3 – 3.5]
Intensive	226	83.0	2.7 [1.7 – 4.6]	147	25.5	0.7 [0.4 – 1.2]
**North-East**						
No	33	59.8	1.0	59	24.2	1.0
Less intensive	32	54.8	0.8 [0.3 – 2.1]	26	58.8	4.5 [1.9 – 10.8]
Intensive	72	73.1	1.8 [1.1 – 4.3]	52	27.4	1.1 [0.5 – 2.9]
**North-South**						
No	55	71.8	1.0	42	58.7	1.0
Less intensive	26	56.4	0.6 [0.2 – 1.4]	25	48.4	0.7 [0.3 – 1.8]
Intensive	67	89.9	3.5 [1.3 – 9.5]	29	37.2	0.5 [0.2 – 1.1]
**North West**						
No	51	79.8	1.0	69	41.6	1.0
Less intensive	29	83.6	1.3 [0.4 – 4.7]	38	51.7	1.5 [0.5 – 4.7]
Intensive	77	71.4	0.7 [0.3 – 1.5]	63	29.7	0.4 [0.1 – 1.6]
**South-East**						
No	35	77.1	1.0	47	22.4	1.0
Less intensive	20	85.0	1.2 [0.3 – 7.1]	15	50.1	3.4 [ 1.1 – 9.2]
Intensive	10	90.1	2.7 [0.3 – 24.3]	3	63.3	NA

PFP: Paid female partners; NPFP: Non-paid female partner

OR: Odds Ratios; 95% CI: 95% Confidence Interval.

NA: Odds Ratio has not been computed because of small cell frequency

Table 6 also indicates that even among those who had no exposure to any HIV prevention program consistent condom use with paid female partners was relatively higher on NS, NW and SE routes than on NE route (NS: 71.8%; NW: 79.8%; SE: 77.1%, NE: 59.8%). The same was true in the case of condom use with non-paid female partners.

Results from multivariate logistic regression analysis confirmed the bi-variate relations between exposure to interventions and consistent condom use after adjusting for the control variables (Table 7). It can be seen that those who were exposed to the intensive program were two times more likely to use condom every time with paid female partners (aOR= 2.4, 95%CI 1.4-4.1). Exposure to less intensive program continued to have positive association with consistent condom use with non-paid female partners but it lost its statistical significance after adjusting for the control variables (aOR = 2.3, 95%CI 0.9 - 3.8).

**Table 7:** Multivariate Analysis: Exposure to interventions and consistent condom use with paid and non-paid female partners, IBBA Round-2 on National Highways

Exposure to intervention and background characteristics	Consistent condom use with PFP	Consistent condom use with NPFP
	
	Adjusted OR [95% CI]	Adjusted OR [95% CI]
**Exposure to HIV prevention program**		
No (Reference category)	1.0	1.0
Less intensive	1.4 [0.8 – 2.5]	2.3 [0.9 – 3.8]
Intensive	2.4 [1.4 – 4.1]	0.6 [0.4 – 1.9]
**Route Categories**		
North-East (Reference category)	1.0	1.0
North- South	1.7 [1.1 – 2.8]	1.7 [0.9 – 3.0]
North-West	1.5 [0.8 – 2.9]	0.9 [0.5 – 1.7]
South-East	1.8 [0.9 – 3.4]	1.0 [0.6 – 1.9]
Current age (years) ^a^	1.0 [0.9 – 1.2]	0.9 [0.8 – 1.1]
**Literacy**		
Illiterate (Reference category)	1.0	1.0
Literate	1.4 [0.7 – 2.2]	1.1 [0.4 – 2.9]
**Marital status**		
Not currently married (Reference category)	1.0	1.0
Currently married	1.2 [0.8 – 1.9]	1.1 [0.6 – 1.7]
Duration of working as truck driver (years) ^a^	0.9 [0.9 – 1.0]	1.0 [ 0.8 – 1.2]
Number of round trips ^a, b^	0.8 [0.8 – 1.1]	0.9 [ 0.8 – 1.1]
**Ownership of truck**		
Respondent (Reference category)	1.0	1.0
Other	0.7 [0.3 – 1.5]	0.6 [0.3 – 1.7]

PFP: Paid female partners; NPFP: Non-paid female partner

OR: Odds Ratios; 95% CI: 95% Confidence Interval

The analysis was done using combined data from all four routes. Adjusted OR were estimated using multivariate logistic regression models.

^a^ Entered as continuous variable in the multivariate logistic regression models.

^b^ Between main cities of operation in past six months

## Discussion

This is the first report of two national level cross-sectional surveys of long-distance truck drivers being used to assess the impact of an HIV prevention intervention in this larger, mobile population. The data shows that overall program has reached about 50% of the long distance truck drivers with considerable variations across the routes. The reported program exposure was found highest north-west route whereas it was found lowest on south-east route. A recent study in India has also concluded low exposure to public funded HIV prevention program among long distance truckers in Andhra Pradesh that falls on the south-east route [29]. Though the proportion of truckers visiting paid female partners remained almost unchanged over time across most of the routes, significant improvements in consistent condom use with paid as well as non-paid female partners were observed. A welcome reduction in the prevalence of Syphilis and HIV was observed at the aggregate level as well as across the routes. These improvements in safer sexual practices and reductions in the STIs were statistically significant in totality as well as in some of the routes.

The data also showed that truck drivers who had sex with paid female partners were significantly more likely to have program exposure, particularly the intensive exposure. On the other hand, those who had sexual contacts with non-paid female partners were more likely to have less intensive exposure. These findings suggests that the program has not just increased its coverage in the targeted population; it has been able to reach those who have sexual contacts outside marriage and hence at more risk of acquiring STI/HIV. This could be due to the two reasons. First, the program purposively targets those who take higher risk. Second, those who have riskier behavior may approach the program to avail information and services [30].

The paper also points out that consistent condom use with paid female partners was higher even among unexposed truckers in NS, NW and SE routes as compared to that in NE route. These points could be explained, at least partly, by attributing this as confounding effect of other parallel interventions among FSWs in several Indian states which are connected through the four route corridors. The NS and NW route corridors connect low HIV prevalence northern Indian states to high HIV prevalence southern and western states whereas the SE route connects the high HIV prevalence southern states to the low/ moderate HIV prevalence eastern states. On the other hand, majority of the NE corridor falls within the low HIV prevalence northern states and partially into the low/ moderate HIV prevalence eastern states [2,31]. Due to higher prevalence of STI/HIV, the western and southern states have been receiving intensive intervention programs (including condom social marketing at ‘hot-spots’ where the commercial sex takes place) among all the high-risk groups, especially FSWs whereas the northern states are known to have low intensive intervention programs among high-risk groups [22,23,3]. The decision on using condom use in a commercial sex is not just a trucker’s own behavior rather it represents behavior of two individuals - trucker and FSW. Thus, besides the effect of truckers’ intervention, higher consistent condom use among respondents traveling on NS, NW and SE routes reflects the effect of interventions targeted towards FSWs. On the other hand, truckers plying on the NE route usually interact with FSWs who do not receive any program of high intensity; hence the consistent condom among unexposed truckers remained at lower level. The possibility that the simultaneous implementation of interventions targeted towards both FSWs and their clients may increase the condom use rates in commercial sex encounters has also been discussed elsewhere [33]. Studies in other settings have shown declines in client STI prevalence as a result of interventions directed towards FSWs [34,35]. Though it is not possible to separate out the effect of several simultaneous interventions, the large difference between the consistent condom uses among unexposed and those having intensive exposure in NE route may be largely attributed to the intervention programs among truckers. However even on this route, we do not deny the possible effects of other programs.

In summary, truckers who had intensive coverage were more likely to use condom consistently with paid female partners. This association was significant even after adjusting the possible confounding effects from socio economic and work related characteristics. This means, once exposed to the intensive program truckers were more likely to adopt safe sexual practices irrespective of important characteristics such as age, literacy, marital status, duration of working as truck drivers, ownership of truck, the routes on which they usually travel and number of round trips. This establishes strong positive effect of intensive exposure with safe sexual practices. It indicates that an integrated intensive large scale intervention can change beliefs and behavior towards safe sexual practices among long distance truckers whereas interventions among truckers that focused on five repeated in-depth interactions with programme staff has been reported to have limited effectiveness [36]. At the same time, the lack of equal success among those who have sex with non-paid female partners is certainly a challenge for the intensive program. Moreover, still about half or less of the targeted population had no exposure of any kind across the four routes. This demands continuing efforts with same intensity to increase the coverage in overall population as well as to achieve universal coverage among those who take greater risk.

## Conclusions

The paper concludes that there is an overall improvement in the safe sexual practices along with the increasing program exposure among long distance truck drivers in the country. The study summarizes that the program has been able to reach at those truckers who took higher risks and once exposed to intensive program these high-risk truckers were more likely to follow safe sexual practices by using condom every time in all commercial sex acts.. The paper also indicates towards the possible contribution of interventions targeted towards FSWs in bringing safe sexual practices among truckers. Realizing that almost half of the truckers have no exposure to any program, we recommend that the intervention programs must be continued with same intensity.

## List of abbreviations used

AIDS: Acquired immune deficiency syndrome; aOR: Adjusted Odds Ratio; aRRR: Adjusted Relative Risk Ratio; CI: Confidence Interval; FSW: Female Sex Workers; HIV: Human Immunodeficiency Virus; IBBA: Integrated Behavioral and Biological Assessment; LDTD: Long Distance Truck Drivers; NE: North-East; NPFP: Non-paid Female Partners; NS: North-South; NW: North-West; OR: Odds Ratios; PFP: Paid Female Partners; RPR: Rapid Plasma Regain; RRR: Relative Risk Ratio; SE: South- East; STI: Sexually Transmitted Infections; TPHA: Treponema Pallidum Hemagglutination Assay; TSL: Transshipment Location.

## Competing interests

The authors have no financial benefits or competing interests related to this submitted work.
